# Features of “All LNA” Duplexes Showing a New Type of Nucleic Acid Geometry

**DOI:** 10.1155/2012/156035

**Published:** 2012-05-14

**Authors:** Charlotte Förster, André Eichert, Dominik Oberthür, Christian Betzel, Reinhard Geßner, Andreas Nitsche, Jens P. Fürste

**Affiliations:** ^1^Institut für Chemie und Biochemie, Freie Universität Berlin, 14195 Berlin, Germany; ^2^Chirurgische klinik II—Visceral, Transplantations-, Thorax- und Gefäßchirurgie, Universitätsklinik Leipzig, Liebigstraße 20, 04103 Leipzig, Germany; ^3^Laboratory of Cell Biology and Howard Hughes Medical Institute, The Rockefeller University, 1230 York Avenue, New York, NY 10065, USA; ^4^Laboratory for Structural Biology of Infection and Inflammation, Institute of Biochemistry and Molecular Biology, University of Hamburg, c/o DESY, 22603 Hamburg, Germany; ^5^Zentrum für Biologische Sicherheit 1, Robert Koch Institut, Nordufer 20, 13353 Berlin, Germany

## Abstract

“Locked nucleic acids” (LNAs) belong to the backbone-modified nucleic acid family. The 2′-*O*,4′-*C*-methylene-**β**-D-ribofuranose nucleotides are used for single or multiple substitutions in RNA molecules and thereby introduce enhanced bio- and thermostability. This renders LNAs powerful tools for diagnostic and therapeutic applications. RNA molecules maintain the overall canonical A-type conformation upon substitution of single or multiple residues/nucleotides by LNA monomers. The structures of “all” LNA homoduplexes, however, exhibit significant differences in their overall geometry, in particular a decreased twist, roll and propeller twist. This results in a widening of the major groove, a decrease in helical winding, and an enlarged helical pitch. Therefore, the LNA duplex structure can no longer be described as a canonical A-type RNA geometry but can rather be brought into proximity to other backbone-modified nucleic acids, like glycol nucleic acids or peptide nucleic acids. LNA-modified nucleic acids provide thus structural and functional features that may be successfully exploited for future application in biotechnology and drug discovery.

## 1. Introduction

Modified nucleic acids have great potential for applications in oligonucleotide-based drug design. As natural RNA and DNA molecules are highly sensitive towards nuclease digestion and often possess low thermal stability, great effort has been made to design nucleic acid modifications that stabilize RNA or DNA while simultaneously maintaining the overall Watson-Crick base pairing ability. Modified nucleic acids are indispensable for future applications comprising diagnostic and clinical approaches like the use of aptamers or the siRNA technology.

Extensive and challenging experiments and investigations have been undertaken to develop nucleotide analogues that maintain the overall A-RNA-type conformation and N-type sugar puckering, as such modifications are likely to allow the substitution of RNA without large changes in functionality. Considerable effort has been made in the synthesis and characterization of 2′-*O*-methyl-RNAs [1], 2′-F-RNAs [2], phosphoramidate-RNAs [3], and the “locked” nucleic acid family [4]. By using locked nucleotide building blocks containing the 2′-*O*,4′-*C*-methylene-**β**-D-ribofuranose (LNA) modification, a significant increase in thermostability can be observed in accordingly substituted RNAs. For example, the melting temperature of modified RNA helices can be increased by +2 to +10°C per LNA monomer substitution.

To understand the stabilizing effects of LNA-substituted RNAs, numerous structural investigations have been performed during the past years to investigate their conformation in detail. These studies provided insights in the local geometric parameters of mix-mer LNA-RNA helices and of LNA-RNA heteroduplexes. The 2′-*O*,4′-*C*-methylene-**β**-D-ribofuranose LNA-RNA mix-mer duplexes maintain mainly the overall A-type nucleic acid conformation [5]. On the other hand, the 2′-*O*,4′-*C*-**α**-L-ribofuranose LNA modification is used in DNA substitution, as this modification preserves the overall B-type nucleic acid geometry of DNA [6]. Thus, there are two powerful nucleotide modifications with great potential in drug design, the 2′-*O*,4′-*C*-methylene-**β**-D-ribofuranose nucleotides (LNA) for RNA substitution and the 2′-*O*,4′-*C*-methylene-**α**-L-ribofuranose nucleotides to modify DNA.

The structure of heteroduplexes, consisting of one fully modified LNA strand hybridized to either RNA or to DNA, revealed the following: the RNA conformation is maintained upon hybridizing a 2′-*O*,4′-*C*-methylene-**β**-D-ribofuranose LNA strand to RNA, whereas a mixed N- and S-type sugar puckering is induced by hybridizing a 2′-*O*,4′-*C*-methylene-**β**-D-ribofuranose LNA to DNA [7]. The B-type conformation is maintained by using a 2′-*O*,4′-*C*-methylene-**α**-L-ribofuranose LNA strand targeted to DNA [6]. It is generally accepted that the *2′*-*O*,4′-*C*-methylene-**β**-D-ribofuranose “locks” the LNA in the C3′-endo conformation. This approach is used to direct the geometry of the phosphate backbone in a manner to orient the duplex towards a more efficient base stacking.

Even though the 2′-*O*,4′-*C*-methylene-**β**-D-ribofuranose LNA-RNA mix-mer duplexes maintain the overall A-type nucleic acid conformation, molecular dynamics simulations [8] and a crystal structure [9] of “all” LNA duplexes, consisting exclusively of 2′-*O*,4′-*C*-methylene-**β**-D-ribofuranose building blocks, yielded insights into a novel nucleic acid geometry. An “all LNA” duplex shows alterations in the local and overall helical parameters as compared to natural RNA and can rather be compared to other modified nucleic acids, like glycol nucleic acids (GNAs) [10], peptide nucleic acids (PNAs) [11], or homo-DNA [12]. An LNA duplex appears as a right-handed, antiparallel helix that maintains the canonical Watson-Crick base pairing and the 2′-exo conformation for all nucleotides. Nevertheless, the LNA duplex shows a considerable decrease in the helical twist, roll and propeller twist, which facilitates a widening of the major groove and a decrease of the minor groove dimensions. These alterations induce a large hollow cave in the middle of the duplex that is obvious in a projection perpendicular to the helical axis. Due to an enlarged helical rise and the unwinding of the helix, which results from the decrease in the twist angle parameters, the LNA duplex possesses an increased helical pitch. The unique nucleic acid geometry of “all LNA” helices apparently induces a more efficient and stable base stacking, which contributes to the higher thermostability of LNAs and LNA-modified nucleic acids. Interestingly, the structure of an RNA/LNA heteroduplex [13] is a geometric intermediate between the RNA and the “all LNA” conformation.

## 2. Material and Methods

### 2.1. Crystallization of the LNA Helices

The 7mer LNA helix was derived from the *E. coli* tRNA^Ser^ isoacceptor with the data base “Compilation of tRNA sequences and sequences of tRNA genes” ID RS 1661 [14] and represents the sequence of the tRNA^Ser^ aminoacyl stem microhelix that has been crystallized previously possessing the sequence 5′-(G-G-U-G-A-G-G-)-3′ and 5′-(C-C-U-C-A-C-C-)-3′ [15]. The LNA helix contained exclusively 2′-*O*,4′-*C*-methylene-**β**-D-ribofuranose building blocks. The base sequence of the RNA was maintained for further comparative studies, except for the U to T and the C to m^5^C exchange used in standard LNA synthesis. The chemically synthesized single strands with the sequences 5′-(G-G-T-G-A-G-G)^L^-3′ and 5′-(m^5^C-m^5^C-T-m^5^C-A-m^5^C-m^5^C)^L^-3′ were purchased from IBA (Göttingen, Germany) with HPLC purification grade. Crystals were grown within 3-4 days using 40 mM sodium cacodylate, pH 5.5, 20 mM cobalt hexamine, 80 mM sodium chloride, 20 mM magnesium chloride, and 10% (v/v) MPD with equilibration against 1 mL 33–41% (v/v) MPD at 21°C using the hanging drop vapour diffusion technique [16].

### 2.2. Diffraction Data Collection and Structure Determination and Refinement

Data collection of the LNA crystals was performed at the ELETTRA synchrotron (Trieste, Italy) beam line XRD-1 at a wavelength of 1.0 Å and a temperature of 100 K. The crystal diffracted up to 1.9 Å resolution [16]. The corresponding tRNA^Ser^-microhelix was measured at the DESY synchrotron (Hamburg, Germany) at a wavelength of 0.8123 Å, 100 K temperature, and diffracted up to 1.2 Å [16]. All data were analyzed and processed using the programs from the HKL-2000 suite [17]. Molecular replacement calculations were performed using the program PHASER [18] within the CCP4i program suite [19]. The RNA structure was solved by molecular replacement using an artificially constructed RNA. The LNA structure was solved by using a model built from the previously solved tRNA^Ser^ microhelix structure but exchanging the riboses by *2′*-*O*,4′-*C*-methylene-**β**-D-ribofuranose residues [9]. Standard LNA nucleotides were used for model building, which comprises the standard U to T and C to m^5^C substitutions in LNA as compared to RNA. Refinement calculations were done applying the program REFMAC [20], and electron density maps were calculated using FFT [21], as implemented in the CCP4i package [19]. Data and refinement statistics are shown in Table 1. The program X3DNA [22] was used to calculate the local and overall geometrical parameters. Structure representations and graphical analysis of helices were performed with the programs COOT [23] and PYMOL [24].

## 3. Results and Discussion

**Figure 1:**
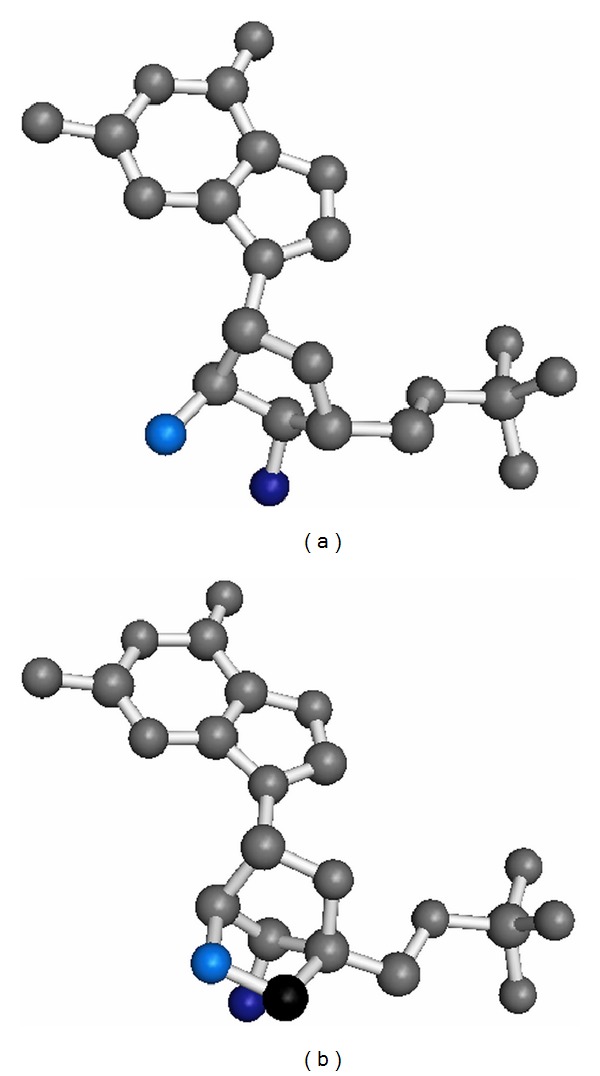
Guanosine monophosphate shown as RNA nucleotide (a) and as LNA nucleotide with the 2′-*O*,4′-*C*-methylene-**β**-D-ribofuranose modification (b). Oxygen atoms are coloured in light blue (2′-oxygens) and dark blue (3′-oxygens), respectively. The additional carbon atom from the methylene group in the LNA is shown in black.

We analysed the crystal structure of a “locked” nucleic acid duplex [9], which contains exclusively 2′-*O*,4′-*C*-methylene-**β**-D-ribofuranose nucleotides (Figure 1), in comparison to the structures of the naturally occurring RNA as well as to other backbone-modified nucleic acids like glycol nucleic acids (GNAs) or peptide nucleic acids (PNAs).

**Figure 2:**
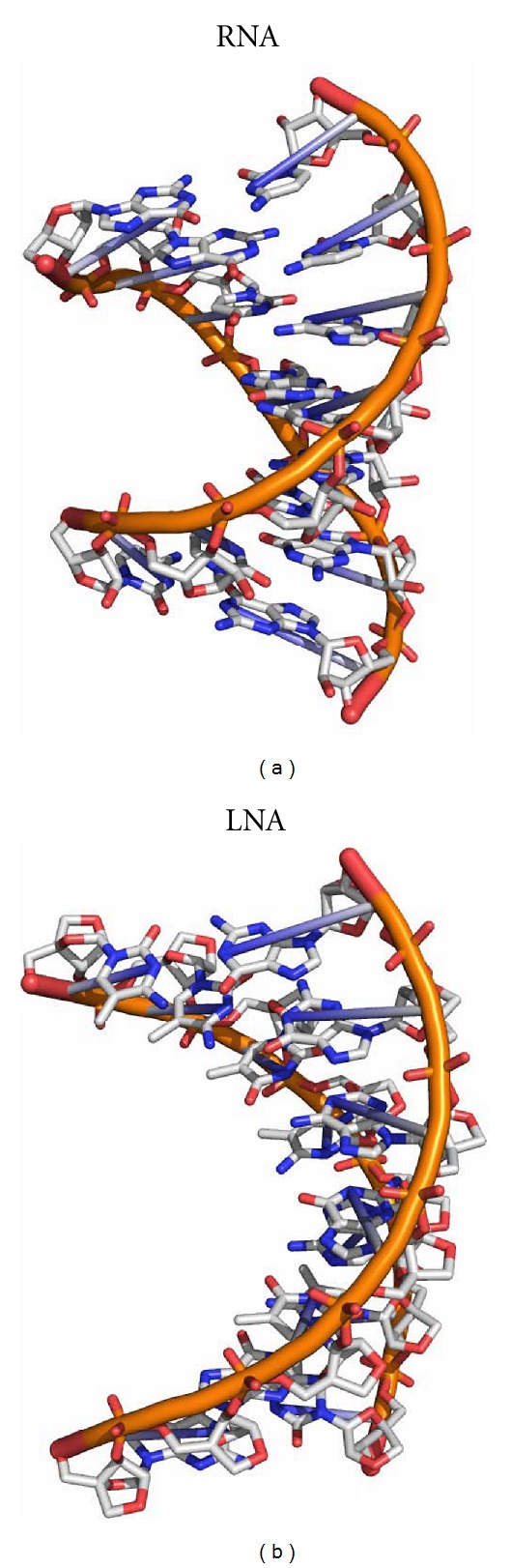
Crystal structure of the RNA duplex r[GGUGAGG]*·*r[CCUCACC] (PDB ID: 3GVN) as compared to the corresponding LNA helix (PDB ID: 2X2Y).

The LNA helix structure reveals a nucleic acid duplex geometry that significantly differs from the canonical A-type RNA structure (Figure 2, Tables 2 and 3). The structure of the LNA duplex appears as a stretched helical ladder with altered local and overall geometric parameters. The observed geometry can be rather compared to that of glycol nucleic acids (GNAs) [25], peptide nucleic acids (PNAs) [26, 27], or homo-DNAs [12]. We detected a notable decrease in several local and overall helical parameters in the LNA helix, like the twist, roll and propeller twist, as compared to a corresponding RNA molecule (Table 2). This results in a widening of the major groove, a decrease in helical winding and an increased helical pitch. The major groove dimensions in the LNA duplex showed values of around 24-25 Å in diameter, as compared to 16 Å observed for the canonical A-RNA duplex. Concomitantly, the minor groove of LNA duplexes is narrower (about 15 Å) than that of standard RNA helices (19 Å). On the other hand, the slide and rise values are slightly increased in the LNA helices. Moreover, the shift of the base pairs in the LNA duplex results in an empty tunnel running through the center of the helix.

In the LNA helix, the low twist angle of 26° and the large pitch of 14 base pairs per turn lead to an unwinding of the duplex, as compared to RNA, which possesses a twist of 32° and a pitch of 11 base pairs per turn. The helical rise of LNA falls into a range of 2.8–3.0 Å, whereas the helical rise in RNA is 2.6 Å. Due to the increased rise and the unwinding of the helix, the LNA possessed an enlarged helical pitch of 39 Å, as compared to 29 Å in RNA helices. The backbone torsion angles resembled the sc^−^, ap^+^, sc^+^, sc^+^ ap, sc^−^, and ap^+^ conformation for the *α*, *β*, *γ*, *δ*, *ε*, *ζ*, and *χ* angles with the sugar puckering being in the 2′-exo conformation. The phosphate-phosphate distances are in the region of 5.6 Å as compared to 6.0 Å for RNA. It is conceivable that the altered helical parameters in LNA duplexes provide an enhancement in nucleotide stacking, leading towards stronger Π-Π interactions of the base pairs.

**Figure 3:**
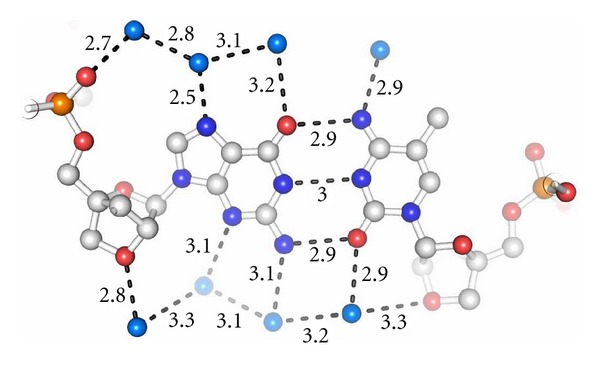
Hydration pattern within the LNA duplex (PDB ID: 2X2): a region of the LNA helix showing the base pair (G4-m^5^C69)^L^. The hydration pattern resembles that known for RNA, as the bridged 2′-oxygen atom in LNA acts as a hydrogen bond acceptor similar to the 2′-oxygen in the hydroxyl group of RNA.

It is well accepted that the extensive hydration of the RNA minor groove plays an important role in the structure/function relationship [28]. As the specific hydration pattern of RNA is governed by the 2′-hydroxyl group, it has been questioned whether the 2′-*O*,4′-*C*-methylene-**β**-D-ribofuranose in LNA allows a comparable hydration as described for RNAs. Therefore, we focussed our investigations on analyzing the arrangement of the solvent molecules surrounding the LNA duplex. We observed that the distribution of water molecules in the LNA minor groove follows the general pattern known for RNA hydration, as the bridged 2′ oxygen atoms in the 2′-*O*,4′-*C*-methylene-**β**-D-ribofuranose moieties serve as hydrogen bond acceptors similar to the 2′-hydroxyl residues in RNA. An example for the LNA hydration is shown in Figure 3.

**Figure 4:**
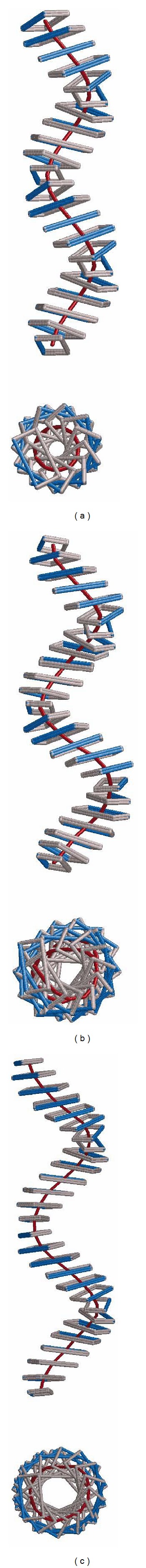
Structure of an idealized RNA helix (a) compared to the RNA/LNA hybrid helix derived from the structure with PDB code 1H0Q (b) and compared to an “all LNA” helix derived from the structure with PDB code: 2X2Q (c) as Calladine-Drew plot. To visualize the overall geometry, the helices were extended to 22 base pairs for RNA, to 25 base pairs for the RNA/LNA, and to 28 base pairs for LNA, respectively [9].

**Figure 5:**
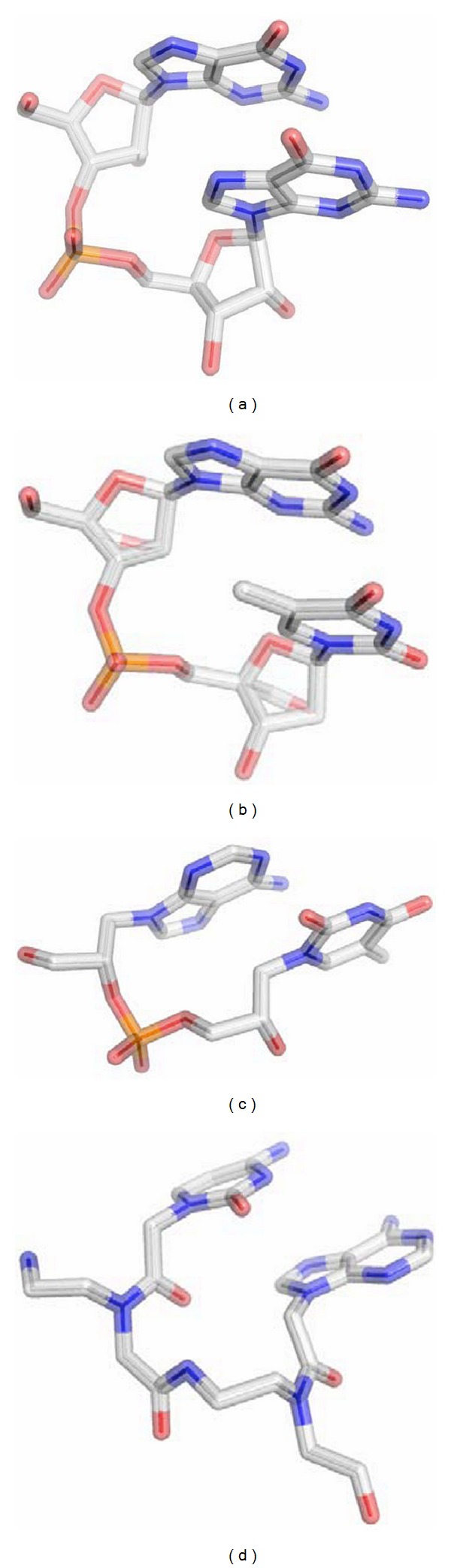
Dinucleotide conformations as observed in RNA (a), LNA with the 2′-*O*,4′-*C*-methylene-**β**-D-ribofuranose modification (b), GNA (c), and PNA (d).

**Figure 6:**
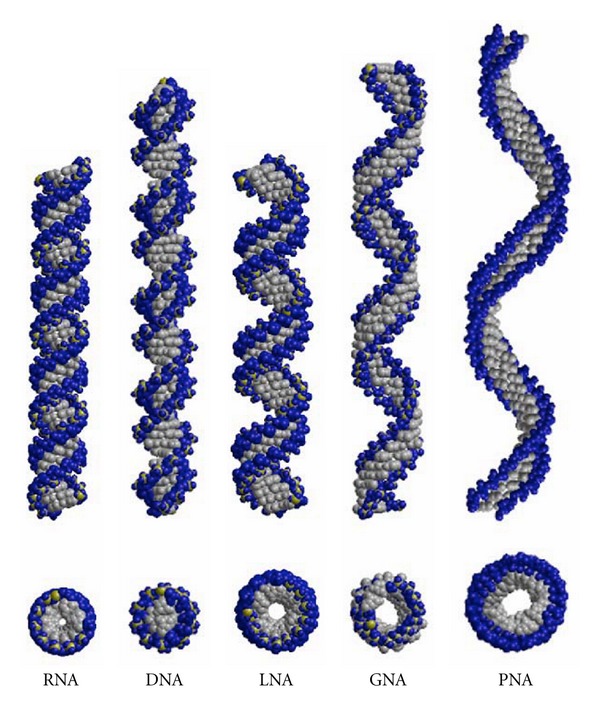
Overall helical structures of natural nucleic acids (RNA and DNA) and of synthetic, modified nucleic acids (LNA, GNA, and PNA). All helices were extended to a total of 46 base pairs. The helices were constructed by using the following structures: DNA (idealized), RNA (idealized), LNA (PDB ID: 2X2Y), GNA (PDB ID: 2JJA), and PNA (PDB ID: 1PUP). Phosphate oxygen atoms are shown in blue, phosphates in yellow, and all other atoms are presented in grey. Top picture shows the side view of the duplexes and the bottom picture presents a projection along the helical axis.

Interestingly, the structure of an RNA/LNA hybrid helix represents a geometric intermediate between RNA and “all LNA” helices. In Figure 4, we present the structure of idealized RNA as compared to the RNA/LNA hybrid (PDB ID: 1H0Q) and to the “all LNA” duplex (PDB ID: 2X2Q) as Calladine-Drew plot. To better visualize the overall geometry in this figure, the helices were extended to 22 base pairs for RNA, to 25 base pairs for RNA/LNA, and to 28 base pairs for LNA showing two full helical turns each [9]. For an overall comparison of A-RNA and B-DNA helices to the conformation of different backbone-modified nucleic acid types, like GNA and PNA (Figure 5), we displayed the selected nucleic acid helices with a total length of 46 base pairs (Figure 6). We illustrate the natural DNA and RNA and the synthetic GNA (PDB ID: 2JJA) and PNA (PDB ID: 1PUP) as compared to the LNA (PDB ID: 2X2Q) duplex structure. The standard A- and B-type nucleic acid conformations are paraphrased by the RNA and DNA helices. The GNA shows the structure of a helical ribbon with only one large minor groove and completely lacks the major groove, which is instead a convex surface [25]. The PNA resembles the helix with a wide and deep major groove concomitant with a narrow and shallow minor groove [26, 27]. The weakly twisted right-handed homo-DNA structure has been described to explain the inability of allo-, altro-, and glucanosyl-nucleotides to form stable base pairing systems (picture not shown) [12]. In the middle of Figure 6 we present the extended structure of the LNA duplex, which represents the unusual geometry, which can rather be brought into vicinity of GNA, PNA, and homo-DNA than to the natural nucleic acid duplexes DNA and RNA. The LNA helix, however, possesses a pitch of 39 Å with 14 base pairs per helical turn and an average rise of 2.8 Å. DNA and RNA show an average pitch of 30 Å and 34 Å, respectively, as compared to mean values of 60 Å, 58 Å, and 53 Å found for GNA, PNA, and homo-DNA. (Figure 6, Table 3, no data shown for homo-DNA). Conclusively, regarding the geometry of the LNA duplex in total, this helix resembles a more natural nucleic acid, when compared to the other backbone-modified duplexes.

As has been reviewed [4], an increase of the melting temperature between +2° to +10°C can be observed per LNA building block added in strands hybridized to RNA. The short 7 bp LNA duplex, derived from the tRNA^Ser^ microhelix for this study, exhibits a melting temperature of above 90°C, whereas the corresponding RNA has a *T*_*m*_ value of 45.0°C [9]. We have previously investigated the melting temperature of another LNA 7mer helix in comparison to its natural RNA counterpart [29]. Similarly, the LNA duplex possesses a *T*_*m*_ value of 84.3°C, whereas its corresponding RNA helix melts at 22.4°C. The drastic shift in thermostability in both LNAs as compared to the RNAs corresponds to an average of 4.5°C per nucleotide building block, which is consistent with the reviewed observations [4]. Thus, the thermostability data and the structure properties of LNAs provide new perspectives for future nucleic acid drug applications, which is an encouraging outlook.

Considering that the increase in *T*_*m*_ values by substituting natural nucleic acids with single or multiple nucleotides by LNA residues seems to be a summative property, the challenge of using LNAs as tools in nucleic acid stabilization becomes obvious. Nearly any natural nucleic acid can be modified by introducing single and multiple LNA building blocks or even complete LNA duplexes, thereby stepwise increasing the thermostability depending on the number of introduced LNA residues. Depending on the particular requirements, any nucleic acid can thus be stabilized at will with little or no loss of function. In this respect, LNA substitution may serve as a reliable method to stabilize nucleic acids, in particular aptamers, for clinical applications.

An upcoming challenge is to stabilize aptamer stem regions by introducing LNA portions without affecting the loop regions that are usually essential for target binding and specificity. Several reports in the literature highlight a forthcoming application of LNA-substituted aptamers with retained or even improved ligand-binding capacity. An exciting example is the use of LNA modifications within hammerhead ribozymes that improve the overall cleaving capacity [30]. In addition, LNA modifications have successfully been introduced into antisense oligonucleotides and DNAzymes that were targeted to functionally selected binding sites and inhibited HIV-1 expression [31]. A third example describes a G-quadruplex thrombin aptamer, which retained the biological activity to a varying extent depending on the nucleotide positions that were LNA modified [32]. These selected reports are a snapshot of numerous studies demonstrating the great potential of LNA substitutions in functional nucleic acids and possible therapeutic applications. The crystal structure of the “all locked” nucleic acid helix contributes to the understanding of the structure/function relationship and the high thermostability of these molecules. In summary LNAs possess an encouraging potential for the development of new stabilized nucleic acids and will promote future applications in diagnostics, drug discovery, and clinical therapy [4].

## Figures and Tables

**Table 1:** Data and refinement statistics of tRNA^Ser^ microhelix and LNA helix [9, 15].

	tRNA^Ser^ microhelix	LNA helix
*Data acquisition *		
Space group	*C*2	*C*2
Cell constants		
*a*, *b*, *c* (Å)	35, 79, 39.13, 31.37	77.91, 40.74, 30.06
**α*, *β*, *γ** (°)	90.00, 111.1, 90.00	90.00, 91.02, 90.00
Resolution (Å)	120–1.20 (1.22–1.20)	80.00–1.90 (1.93–1.90)
*R*_merge_	7.4 (15.4)	7.3 (21.7)
*I*/*σI *	18.7 (1.8)	19.7 (1.0)
Completeness (%)	99.2 (99.1)	98.0 (97.2)
Redundancy	7.1 (8.6)	4.8 (3.8)

*Refinement *		
No. of reflections	12,806	7,382
*R*_work_ /*R*_free_	19.0 (20.1)	22.9 (28.8)
Atoms		
Nucleic acid	293	314
Magnesium	2	1
Cobalt hexamine	—	3
Water oxygens	97 (2 mol/au)	44

Values in parentheses are given for the highest-resolution shell.

**Table 2:** Selected overall helical parameters of the tRNA^Ser^ microhelix structure (PDB ID: 3GVN) compared to the LNA-RNA hybrid (PDB ID: 1H0Q) and the LNA helix (PDB ID: 2X2Y). The two LNA molecules correspond to two LNA helices located in the asymmetric unit of the crystal structure.

	Twist (°)	Rise (Å)	Slide (°)	Roll (°)	*χ*-displacement (Å)	Propeller twist (°)
RNA	32.46	2.64	−1.68	6.61	−4.25	−10.46
tRNA^Ser^ microhelix						

LNA-RNA	29.22	2.65	−2.24	6.07	−5.40	−12.84
hybrid						

LNA						
(molecule A)	25.97	2.81	−2.49	4.08	−6.60	−6.65
tRNA^Ser^ microhelix						

LNA						
(molecule B)	26.13	2.84	−2.47	4.15	−6.47	−7.45
tRNA^Ser^ microhelix						

**Table 3:** Overall helical parameters for natural (RNA and DNA) and modified nucleic acids (LNA, GNA, and PNA).

	Base pairs/helical turn	Twist (°)	Rise (Å)	P-P distance (Å)	Pitch (Å)
RNA	11	32	2.6	6.0	30
DNA	10	36	3.4	7.0	34
LNA	14	26	2.8	5.6	39
GNA	16	22.9	3.8	5.4	60
PNA	18	19	3.2	5.4	58

## References

[1] Manoharan M (1999). 2’-Carbohydrate modifications in antisense oligonucleotide therapy: importance of conformation, configuration and conjugation. *Biochimica et Biophysica Acta*.

[2] Kawasaki AM, Casper MD, Freier SM (1993). Uniformly modified 2′-deoxy-2′-fluoro phosphorothioate oligonucleotides as nuclease-resistant antisense compounds with high affinity and specificity for RNA targets. *Journal of Medicinal Chemistry*.

[3] Gryaznov SM (1999). Oligonucleotide N3'→P5'-phosphoramidates as potential therapuetic agents. *Biochimica et Biophysica Acta*.

[4] Petersen M, Wengel J (2003). LNA: a versatile tool for therapeutics and genomics. *Trends in Biotechnology*.

[5] Petersen M, Bondensgaard K, Wengel J, Peter Jacobsen J (2002). Locked nucleic acid (LNA) recognition of RNA: NMR solution structures of LNA: RNA hybrids. *Journal of the American Chemical Society*.

[6] Nielsen KM, Petersen M, Håkansson AE, Wengel J, Jacobsen JP (2002). alpha-L-LNA (alpha-L-ribo configured locked nucleic acid) recognition of DNA: an NMR spectroscopic study.. *Chemistry*.

[7] Nielsen KE, Rasmussen J, Kumar R, Wengel J, Jacobsen JP, Petersen M (2004). NMR studies of fully modified locked nucleic acid (LNA) hybrids: solution structure of an LNA:RNA hybrid and characterization of an LNA:DNA hybrid. *Bioconjugate Chemistry*.

[8] Pande V, Nilsson L (2008). Insights into structure, dynamics and hydration of locked nucleic acid (LNA) strand-based duplexes from molecular dynamics simulations. *Nucleic Acids Research*.

[9] Eichert A, Behling K, Betzel C, Erdmann VA, Fürste JP, Förster C (2010). The crystal structure of an “All Locked” nucleic acid duplex. *Nucleic Acids Research*.

[10] Schlegel MK, Essen LO, Meggers E (2008). Duplex structure of a minimal nucleic acid. *Journal of the American Chemical Society*.

[11] Rasmussen H, Kastrup SJ, Nielsen JN, Nielsen JM, Nielsen PE (1997). Crystal structure of a peptide nucleic acid (PNA) duplex at 1.7 Å resolution. *Nature Structural Biology*.

[12] Egli M, Pallan PS, Pattanayek R (2006). Crystal structure of homo-DNA and nature’s choice of pentose over hexose in the genetic system. *Journal of the American Chemical Society*.

[13] Petersen M, Bondensgaard K, Wengel J, Peter Jacobsen J (2002). Locked nucleic acid (LNA) recognition of RNA: NMR solution structures of LNA:RNA hybrids. *Journal of the American Chemical Society*.

[14] Sprinzl M, Vassilenko KS (2005). Compilation of tRNA sequences and sequences of tRNA genes. *Nucleic Acids Research*.

[15] Eichert A, Fürste JP, Schreiber A (2009). The 1.2 Å crystal structure of an *E. coli* tRNASer acceptor stem microhelix reveals two magnesium binding sites. *Biochemical and Biophysical Research Communications*.

[16] Behling K, Eichert A, Fürste JP, Betzel C, Erdmann VA, Förster C (2009). Crystallization and X-ray diffraction analysis of an all-locked nucleic acid duplex derived from a tRNASer microhelix. *Acta Crystallographica Section F*.

[17] Otwinowski Z, Minor W (1997). Processing of X-ray diffraction data collected in oscillation mode. *Methods in Enzymology*.

[18] McCoy AJ, Grosse-Kunstleve RW, Storoni LC, Read RJ (2005). Likelihood-enhanced fast translation functions. *Acta Crystallographica Section D*.

[19] Collaborative Computational Project, Number 4 (1994). The CCP4 suite: programs for protein crystallography. *Acta Crystallographica*.

[20] Murshudov GN, Vagin AA, Dodson EJ (1997). Refinement of macromolecular structures by the maximum-likelihood method. *Acta Crystallographica Section D*.

[21] Read RJ, Schierbeek AJ (1988). A phased translation function. *Journal of Applied Crystallography*.

[22] Lu XJ, Olson WK (2003). 3DNA: a software package for the analysis, rebuilding and visualization of three-dimensional nucleic acid structures. *Nucleic Acids Research*.

[23] Emsley P, Cowtan K (2004). Coot: model-building tools for molecular graphics. *Acta Crystallographica Section D*.

[24] DeLano WL (2002). *The PyMOL Molecular Graphics System*.

[25] Schlegel MK, Essen LO, Meggers E (2008). Duplex structure of a minimal nucleic acid. *Journal of the American Chemical Society*.

[26] He W, Hatcher E, Balaeff A (2008). Solution structure of a peptide nucleic acid duplex from NMR data: features and limitations. *Journal of the American Chemical Society*.

[27] Rasmussen H, Kastrup SJ, Nielsen JN, Nielsen JM, Nielsen PE (1997). Crystal structure of a peptide nucleic acid (PNA) duplex at 1.7 Å resolution. *Nature Structural Biology*.

[28] Auffinger P, Westhof E (1998). Hydration of RNA base pairs. *Journal of Biomolecular Structure and Dynamics*.

[29] Förster C, Oberthuer D, Gao J (2009). Crystallization and preliminary X-ray diffraction data of an LNA 7-mer duplex derived from a ricin aptamer. *Acta Crystallographica Section F*.

[30] Christiansen JK, Lobedanz S, Arar K, Wengel J, Vester B (2007). LNA nucleotides improve cleavage efficiency of singular and binary hammerhead ribozymes. *Bioorganic and Medicinal Chemistry*.

[31] Jakobsen MR, Haasnoot J, Wengel J, Berkhout B, Kjems J (2007). Efficient inhibition of HIV-1 expression by LNA modified antisense oligonucleotides and DNAzymes targeted to functionally selected binding sites. *Retrovirology*.

[32] Bonifacio L, Church FC, Jarstfer MB (2008). Effect of locked-nucleic acid on a biologically active G-quadruplex. A structure-activity relationship of the thrombin aptamer. *International Journal of Molecular Sciences*.

